# Sanguisorbae Radix Suppresses Colorectal Tumor Growth Through PD-1/PD-L1 Blockade and Synergistic Effect With Pembrolizumab in a Humanized PD-L1-Expressing Colorectal Cancer Mouse Model

**DOI:** 10.3389/fimmu.2021.737076

**Published:** 2021-09-29

**Authors:** Eun-Ji Lee, Ji Hye Kim, Tae In Kim, Yeon-Ji Kim, Malk Eun Pak, Chang Hyun Jeon, Yeo Jin Park, Wei Li, Young Soo Kim, Jang-Gi Choi, Hwan-Suck Chung

**Affiliations:** Korean Medicine Application Center, Korea Institute of Oriental Medicine, Daegu, South Korea

**Keywords:** PD-1, PD-L1, Sanguisorbae Radix, cancer immunology, humanized PD-1 mice, tumor-infiltrating CD8^+^ T cell

## Abstract

Immune checkpoints such as programmed death-1 (PD-1) have been proven as antitumor targets by enhancing cytotoxic T cell activity. All immune checkpoint blockades are antibody therapeutics that have large size and high affinity, as well as known immune-related side effects and low responses. To overcome the limitation of antibody therapeutics, we have explored PD-1/PD-L1 (programmed death-ligand 1) blockades in traditional oriental medicine, which has a long history but has not yet studied PD-1/PD-L1 blockades. Sanguisorbae Radix extract (SRE) blocked PD-1 and PD-L1 binding in competitive ELISA. SRE effectively inhibited the PD-1/PD-L1 interaction, thereby improving T cell receptor (TCR) signaling and the NFAT-mediated luciferase activity of T cells. SRE treatment reduced tumor growth in the humanized PD-L1 MC38 cell allograft humanized PD-1 mouse model. Additionally, the combination of SRE and pembrolizumab (anti-PD-1 antibody) suppressed tumor growth and increased infiltrated cytotoxic T cells to a greater extent did either agent alone. This study showed that SRE alone has anticancer effects *via* PD-1/PD-L1 blockade and that the combination therapy of SRE and pembrolizumab has enhanced immuno-oncologic effects.

## Introduction

Colorectal cancer (CRC) is the third most common cancer in terms of morbidity and mortality around the world (1). The treatment of advanced CRC has applied chemotherapy, including 5-fluorouracil, oxaliplatin, and irinotecan, and targeted therapy, including bevacizumab and cetuximab, but these agents have resistance, dose-limiting side effects, and are strongly toxic to normal cells (2, 3). As an improvement over these agents, immunotherapy has been developed as an effective treatment, increasing the strength of the immune system against malignant tumors in patients with CRC along with microsatellite types or mismatch-repair deficiency (4). In immunotherapy, immune checkpoint inhibitors (ICIs) targeting PD-1 or PD-L1 in several human cancers have recently been demonstrated. To date, six ICIs have been approved by the FDA, including pembrolizumab, nivolumab, cemiplimab, atezolizumab, avelumab, and durvalumab (5). The programmed death-1/programmed death-ligand 1 (PD-1/PD-L1) signaling axis can be important to tumor survival and development (6). The PD-L1 transmembrane protein is known to express on the surface of CRC, and its binding with PD-1 leads to the escape of cancer cells from immune-mediated destruction, thereby enhancing cancer cell growth (7). PD-1 is a crucial immune checkpoint molecule and is mainly expressed on the tumor-infiltrating T lymphocytes (TILs), including CD4^+^ T cells (helper T cells) and CD8^+^ T cells (cytotoxic T lymphocytes, CTLs) (8). TILs activated by the blocking of the PD-1/PD-L1 interaction release antitumor cytokines containing interleukin-2 (IL-2) and interferon (IFN)-γ, which indirectly help the immune system fight cancer cells (6). In particular, CTLs can directly induce cancer cell death by releasing lytic granules, such as the perforin (PRF) protein, which punches holes in the cancer cell membrane (9). Immunomodulatory activity according to the blocking of the PD-1/PD-L1 interaction enhances the antitumor activity of CTLs in the tumor microenvironment.

Recently, anti-PD-1 medications, such as pembrolizumab and nivolumab, in combination with chemoradiotherapy or targeted therapy has been reported to effectively inhibit the PD-1/PD-L1 interaction in CRC (10). Although these clinical therapies have the advantage of increasing survival rate in patients with CRC, their strong toxicity causes several adverse effects, they have a long half-life, and are expensive to produce (3). Use of novel supplementary therapies, including those from natural sources, is therefore imperative. Compared to clinical drugs, these are rapidly absorbed in the human body, and doses can be easily adjusted, raising the expectation of cure by enhancing patients’ long-term survival rate (11). Medicinal herbs containing abundant bioactive ingredients have been utilized as CRC therapeutics to overcome the toxicity and resistance of clinical anticancer drugs (12). Previous investigations regarding the targeting of PD-1/PD-L1 have focused on antibody drugs for CRC immunotherapy but not on known traditional medicinal plants. To discover the more effective CRC immunotherapeutics, we focused on natural products to find combinatorial ICIs with potent synergistic efficacies on the PD-1/PD-L1 interaction.

Sanguisorbae Radix (SR), the dried root of *Sanguisorba officinalis*, also known as great burnet, is a traditional herbal medicine used to treat diarrhea, chronic intestinal inflammation, duodenal ulcers, and internal hemorrhage (13). Recently, multiple studies have reported its diverse pharmacological actions, including antiallergic, anti-inflammatory, antiobesity, and anticancerous (14–17). Although several studies have shown that SR extract (SRE) suppresses both *in vivo* and *in vitro* CRC growth (18, 19), to our knowledge, no literature has reported the antitumor effects of SRE on targeting the PD-1/PD-L1 signaling axis.

Ongoing screening of PD-1/PD-L1 inhibitor from herbal medicine, we found that SRE is a potent inhibitor of PD-1/PD-L1 interaction by *in vitro* competitive ELISA and cell-based luciferase assay. Additionally, we established the antitumor effect of SRE in combination with anti-PD-1 antibodies using a humanized PD-L1 MC38 CRC cell-bearing humanized PD-1 knockin mouse model. That is distinguished studies for CRC immunotherapies targeting human PD-1/PD-L1 in the animal model. Based on this investigation, we proposed a novel combination strategy to improve the effectiveness of immunotherapy by using SRE in cancer patients.

## Materials and Methods

### Preparation of Plant Materials

SR was supplied by the National Development Institute of Korean Medicine (NIKOM, Gyeongsan, Korea). The dried whole plant (2.0 kg) was extracted with 5 L of 70% ethanol for 1 hour, three times. The extract was percolated with filter paper (3 mm; Whatman PLC, Kent, UK), condensed using a rotary evaporator (Buchi, Swiss), and lyophilized using a freeze dryer (Eyela, Japan). The extract powder (285.41 g; yield 14.27%; abbreviated as SRE) was dissolved in 50% dimethyl sulfoxide for stock solution (100 mg/mL) and diluted with culture medium for *in vitro* assay.

### PD-1/PD-L1 Competitive ELISA

PD-1/PD-L1 competitive ELISA (#72005, BPS Bioscience, San Diego, CA, USA) was performed as per the manufacturer’s protocol. As a positive control, an anti-PD-1 neutralizing antibody (#71120) was purchased from BPS Bioscience. Briefly, recombinant hPD-L1 protein (#71104, BPS Bioscience) was coated on the plates (0.32 cm^2^, #3917, Corning, New York, NY, USA) at 1 μg/mL with phosphate-buffered saline (PBS, pH 7.4) and incubated overnight at 4°C. The plates were washed with PBS containing 0.05% Tween 20 (PBS-T) and blocked with PBS-T containing 2% (w/v) bovine serum albumin for 1 hour at room temperature (RT). The biotinylated hPD-1 (#71109, BPS Bioscience) of 0.5 μg/mL was added to each well and incubated for 2 hours at RT. The horseradish peroxidase (HRP)-conjugated streptavidin (#554066, BD Biosciences, San Jose, CA, USA) of 0.2 μg/mL was added to each well and incubated for 1 hour at RT. The relative chemiluminescence was measured using a SpectraMax L microplate reader (Molecular Devices, San Jose, CA, USA).

### Cell Culture

Recombinant Jurkat T cells expressing human PD-1 and NFAT reporter gene (#60535, hPD-1/NFAT Jurkat T cells) and recombinant CHO-K1 cells expressing human PD-L1 and T cell receptor (TCR) activator (#60536, hPD-L1/TCR CHO-K1 cells) were purchased from BPS Bioscience. The hPD-1/NFAT Jurkat T cells were maintained in Roswell Park Memorial Institute (RPMI) 1640 medium supplemented with 10% (v/v) heat-inactivated fetal bovine serum (FBS) and antibiotics (100 U/mL penicillin and 100 μg/mL streptomycin). The hPD-L1/TCR CHO-K1 cells were maintained in Ham’s F-12 medium supplemented with 10% (v/v) heat-inactivated FBS and antibiotics. These cells were cultured in a complete medium with Geneticin (1 mg/mL) and Hygromycin B (200 μg/mL) to maintain stable cells containing genetic constructs. MC38 cells expressing human PD-L1 (hPD-L1 MC38 cells), derived from C57BL/6 murine colorectal adenocarcinoma, were purchased from Shanghai Model Organisms Center, Inc. (Shanghai, China). The hPD-L1 MC38 cells were maintained in Dulbecco’s Modified Eagle Medium supplemented with 10% (v/v) heat-inactivated FBS, antibiotics, and Hygromycin B (50 μg/mL). The cells were incubated in a humidified incubator at 37°C under a 5% CO_2_ atmosphere before the experiments. These solutions for cell culture were purchased from Hyclone Laboratories, Inc. (GE Healthcare Life Sciences, Chicago, IL, USA).

### Cell Counting Kit-8 Assay

The cytotoxic effect of SRE was examined using CCK (#CK04, Dojindo Molecular Technologies, Inc., Rockville, MD, USA) assay. Briefly, the cells (1 × 10^4^ cells/0.32 cm^2^) were cultured in the indicated concentrations (0–400 μg/mL) of SRE at 37°C. CCK solution (10 μL) was added into each well, and the culture plates were incubated for 2 hours at 37°C. The absorbance of the formazan products in the cell culture medium was measured using a SpectraMax i3 microplate reader (Molecular Devices, San Jose, CA, USA) at 450 nm.

### PD-1/PD-L1 Blockade Bioassay

PD-1/PD-L1 blockade bioassay (#J4011, Promega, Madison, WI, USA) was performed following the manufacturer’s protocol. Briefly, hPD-L1/TCR CHO-K1 cells (1 × 10^4^ cells/0.32 cm^2^) as target cells were cocultured with the hPD-1/NFAT Jurkat T cells (2 × 10^4^ cells/0.32 cm^2^) as effector cells and the indicated concentrations of SRE (0–50 μg/mL) or anti-PD-1(0–0.5 μg/mL) neutralizing antibody for 24 hours at 37°C. Bio-Glo™ Assay reagent was added into each well, and the chemiluminescence of culture plates was measured using a SpectraMax L microplate reader.

### Humanized PD-1 Mice

Genetically modified C57BL/6J mice that express human full-length PD-1 protein (humanized PD-1 mice) were purchased from Shanghai Model Organisms Center (Shanghai, China). Mice were granted free access to a standard diet with drinking water before the experiment. All mice were housed under specific-pathogen-free facilities of the Korea Institute of Oriental Medicine (KIOM). All mice were housed in laboratory cage rack systems maintained at a constant temperature (22 ± 1°C) and humidity (50 ± 5%) under a 12-hour dark/light cycle. All experimental procedures followed the Guidelines for the Care and Use of Laboratory Animals of the National Institutes of Health of Korea and were approved by the Institutional Animal Care and Use Committee of KIOM (approval number KIOM-D-20-073).

### Isolation and Culture of Murine Splenocytes and Tumor-Infiltrating CD8^+^ T Cells

Splenocytes were isolated from the spleens of hPD-L1 MC38 cell-bearing hPD-1 knockin mice. The single-cell suspension of splenocytes was obtained by first filtering through a 100-μm and 40-μm cell strainer (SPL Life Sciences, Pocheon, Korea) and then adding ammonium-chloride-potassium lysing buffer (Lonza, Basel, Switzerland) to remove red blood cells. Tumor-infiltrating CD8^+^ T cells were isolated from tumor tissues of hPD-L1 MC38 cell-bearing hPD-1 knockin mice. The single-cell suspension of hPD-L1 MC38 tumor tissues was obtained by digesting the tissues with collagenase (0.5 mg/mL collagenase IV) for 1 hour at 37°C and then filtering through 100-μm and 40-μm cell strainer. The hPD-L1 MC38 tumor-infiltrating CD8^+^ T cells was purified by immunomagnetic negative selection (#19853, STEMCELL Technologies, Inc., Vancouver, Canada). The cells were cultured in RPMI 1640 medium supplemented with 10% (v/v) heat-inactivated FBS and antibiotics.

### T Cell-Mediated Killing of Cancer Cells by Coculture System

Murine lymphocytes and tumor-infiltrating CD8^+^ T cells (1 × 10^6^ cells/9.5 cm^2^) as effector cells were activated with Dynabeads T-Activator CD3/CD28 (Life Technologies, Carlsbad, CA, USA) for 72 hours at 37°C. Murine hPD-L1 MC38 cells were stained with CellTrace™ Far Red Cell Proliferation Kit (Thermo Fisher Scientific, Waltham, MA, USA). The hPD-L1 MC38 cells (5 × 10^4^ cells/1.9 cm^2^) as target cells were treated with IFN-γ (10 ng/mL) for triggering reactive expression of PD-L1 for 24 hours at 37°C. The hPD-L1 MC38 cells were cocultured with the activated CD8^+^ T cells (2.5 × 10^5^ cells/1.9 cm^2^) at an effector cell-to-target cell ratio of 5:1 or with splenocytes (5 × 10^5^ cells/1.9 cm^2^) at an effector cell-to-target cell ratio of 10:1 and the indicated concentrations (0–50 μg/mL) of SRE for 72 hours at 37°C. After 72 hours, the plates were washed with PBS, the remaining attached live cancer cells were stained with crystal violet solution and measured using a SpectraMax i3 microplate reader at 540 nm. The cocultured hPD-L1 MC38 cells were observed using fluorescence microscopy (Olympus, Tokyo, Japan) and analyzed using a flow cytometer (Beckman Coulter, Inc., Brea, CA, USA).

### LDH Cytotoxicity Assay

The lactate dehydrogenase (LDH) liberated from the target cells *via* effector cells was measured using LDH cytotoxicity assay (#ab65393, Abcam, Cambridge, UK). Briefly, the cell culture medium was mixed with WST Substrate Mix and incubated for 30 min at RT. The reaction was stopped by adding a stop solution, and the absorbance of the formazan products was measured using a SpectraMax i3 microplate reader at 450 nm.

### IL-2 Measurement Assay

The amount of IL-2 released by activated T cells in the cell coculture supernatants was measured using a sandwich ELISA (#555148, BD Biosciences) according to the manufacturer’s protocol. Briefly, an anti-mouse IL-2 monoclonal antibody was coated on the plates (0.32 cm^2^, #3590, Corning) with 0.1 M sodium carbonate (pH 9.5) and incubated overnight at 4°C. The plates were washed with PBS-T and blocked with PBS containing 10% (w/v) FBS for 1 hour at RT. The biotinylated IL-2 antibody and streptavidin-HRP were added to each well and incubated for 1 hour at RT. The relative absorbance was measured using a SpectraMax i3 microplate reader at 450 nm.

### PRF1 Measurement Assay

The concentrations of PRF1 released by activated T cells in the cell coculture supernatants were quantified using a sandwich ELISA (NBP3-00452, Novus Biologicals, Centennial, CO, USA) according to the manufacturer’s protocol. Briefly, the standard and samples were added to the coating plates and incubated for 90 min at 37°C. The biotinylated PRF1 and HRP-streptavidin were added to each well, then incubated for 1 hour at 37°C. The relative absorbance was measured using a SpectraMax i3 microplate reader at 450 nm.

### Tumor Allograft and SRE Treatment

Human PD-L1 MC38 cells (3 × 10^5^ cells/200 μL PBS) were injected into the dorsal subcutaneous skin of C57BL/6J humanized PD-1 knockin mice. Tumor growth was monitored and tumor size was measured using digital calipers (Hi-Tech Diamond, Westmont, IL, USA), and tumor volume was calculated according to the formula (length × width^2^)/2. The tumor volumes reached 20 mm^3^ (day 10), and mice were randomized into groups of six animals per group. The vehicle (PBS) group and SRE-treated groups were orally administered 100 mg/kg and 300 mg/kg of SRE in 100 μL PBS/20 g once daily using oral zonde for 17 days, respectively. The anti-PD-1 antibody-treated group was administered 2.5 mg/kg of pembrolizumab in 100 μL PBS/20 g *via* intraperitoneal injection on days 1, 4, 8, and 15. All mice were euthanized for analysis 18 days after treatment.

### Blood Biochemistry

Blood sera were collected *via* cardiac puncture of humanized PD-1 mice treated with SRE and anti-PD-1 antibodies using blood-collection tubes (#365967, BD Biosciences). The levels of AST, ALT, blood urea nitrogen (BUN), and creatinine were analyzed using biochemical analyzer XL 200 (Erba Lachema s.r.o, Mannheim, Germany).

### Immunohistochemistry

For immunohistochemical analysis, the tumor tissues were fixed with 10% formalin in PBS and embedded in paraffin. The paraffin sections were incubated with a primary antibody against the CD8 (#98941, Cell Signaling Technology, Danvers, MA, USA) and PRF (#31647, Cell Signaling Technology). The tissue slides were visualized *via* DAKO EnVision kit (#K5007, DAKO, Jena, Germany). The sections were counterstained with hematoxylin. Hematoxylin-eosin stain was performed for histopathological examination of tumor tissues. Images were observed using an Olympus BX53 microscope and XC10 microscopic digital camera (Tokyo, Japan).

### Statistical Analysis

The data are presented as the mean ± standard deviation and were analyzed using GraphPad Prism (GraphPad Software, Inc., La Jolla, CA, USA). The difference of mean values was analyzed by one-way ANOVA, followed by Tukey’s *post hoc* test, which was used for comparisons between multiple groups, as indicated. Differences with a *p*-value <0.05 were considered significant. All experiments except those in the animal studies were conducted on at least three independent occasions.

## Results

### SRE Blockade of PD-1/PD-L1 Interaction

**Figure 1:**
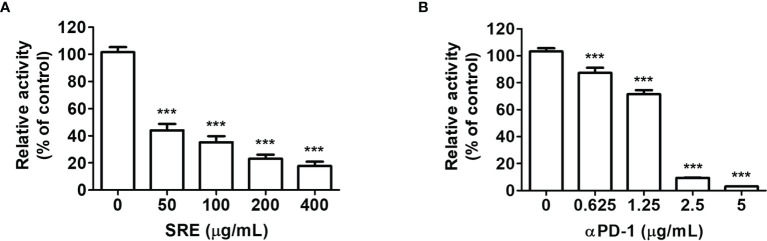
Sanguisorbae Radix extract (SRE) blockade of programmed death-1/programmed death-1 ligand (PD-1/PD-L1) interaction using competitive ELISA. The competitive ELISA was performed using the PD-1/PD-L1 inhibitor screening assay kit. The indicated concentrations of SRE **(A)** and anti-PD-1 antibodies (αPD-1) **(B)** were treated on plates coated with PD-L1, then incubated with biotin-labeled PD-1. Data are presented as the mean ± SD. ***p < 0.001 compared to the control.

To investigate the effect of SRE on PD-1/PD-L1 blockade, we experimented with competitive PD-1/PD-L1 ELISA-binding assays. The results showed that SRE blocked the binding of PD-1 to PD-L1 in a dose-dependent manner (**Figure 1A**). In controls, anti-PD-1 antibodies also inhibited PD-1/PD-L1 interaction in a concentration-dependent manner (**Figure 1B**). The 50% inhibitory concentration (IC_50_) values of SRE and anti-PD-1 antibodies were 50.10 ± 15.14 μg/mL and 1.49 ± 20.59 μg/mL, respectively.

### SRE Blockade of PD-1/PD-L1 in Luciferase Assay

**Figure 2:**
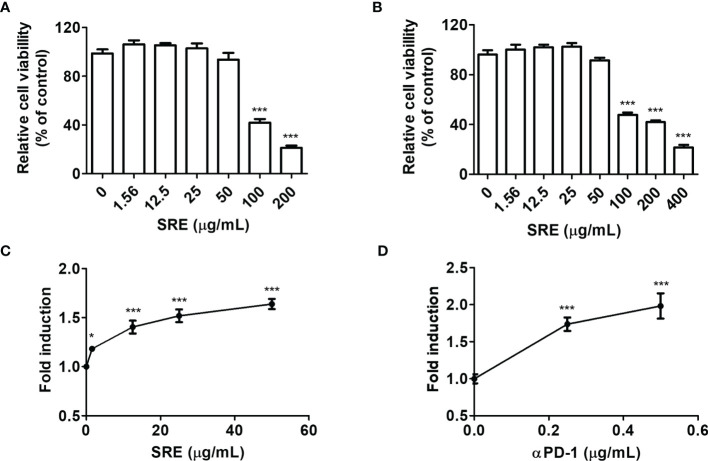
SRE blockade of PD-1/PD-L1 interaction in coculture cell-based luciferase assay. **(A, B)** Cytotoxicity assay performed using Cell Counting Kit-8 (CCK) assay. The hPD-1/NFAT Jurkat T cells **(A)** and hPD-L1/TCR CHO-K1 cells **(B)** after treatment with SRE for 24 hours. **(C, D)** The PD-1/PD-L1 blockade bioassay was performed using the Bio-Glo™ luciferase assay system. After addition of hPD-1/NFAT Jurkat T cells and SRE **(C)** and anti-PD-1 antibodies (αPD-1) **(D)**, hPD-L1/TCR CHO-K1 cells were seeded for 20 hours. Data are presented as the mean ± SD. *p < 0.05 and ***p < 0.001 compared to the control.

To elucidate the effect of SRE on TCR activation, we conducted coculture systems using human PD-1-expressing Jurkat T cells that expressed NFAT-derived luciferase reporter (hPD-1/NFAT Jurkat cells) and human PD-L1-expressing aAPC/CHO-K1 cells designed to activate cognate TCR (hPD-L1/TCR CHO-K1 cells). The cell culture model was established to evaluate the effect of PD-1/PD-L1 blockade (20). To examine the cytotoxic effects of SRE, hPD-1 effector cells and hPD-L1/TCR CHO-K1 cells were treated with the indicated concentration (0–50 μg/mL) of SRE for 24 hours. SRE had no cytotoxic effect on cells up to the concentration of 50 μg/mL (**Figures 2A, B**). The 50% cytotoxic concentration (CC_50_) values of SRE on the hPD-1/NFAT Jurkat cells and hPD-L1/TCR CHO-K1 cells for 24 hours were 97.03 ± 11.51 μg/mL and 139.45 ± 11.49 μg/mL, respectively. The effect of SRE in coculture cell model systems using a PD-1/PD-L1 blockade bioassay was examined. The 50% effective concentration (EC_50_) values of SRE and anti-PD-1 antibodies were 4.974 ± 0.04 μg/mL and 0.239 ± 0.08 μg/mL, respectively (**Figures 2C, D**). These findings suggest that SRE effectively inhibited the PD-1/PD-L1 interaction, thereby improving TCR signaling and NFAT-mediated luciferase activity of T cells.

### Enhancement of T Cell-Mediated Killing of Cancer Cells by SRE Blockade of PD-1/PD-L1

**Figure 3:**
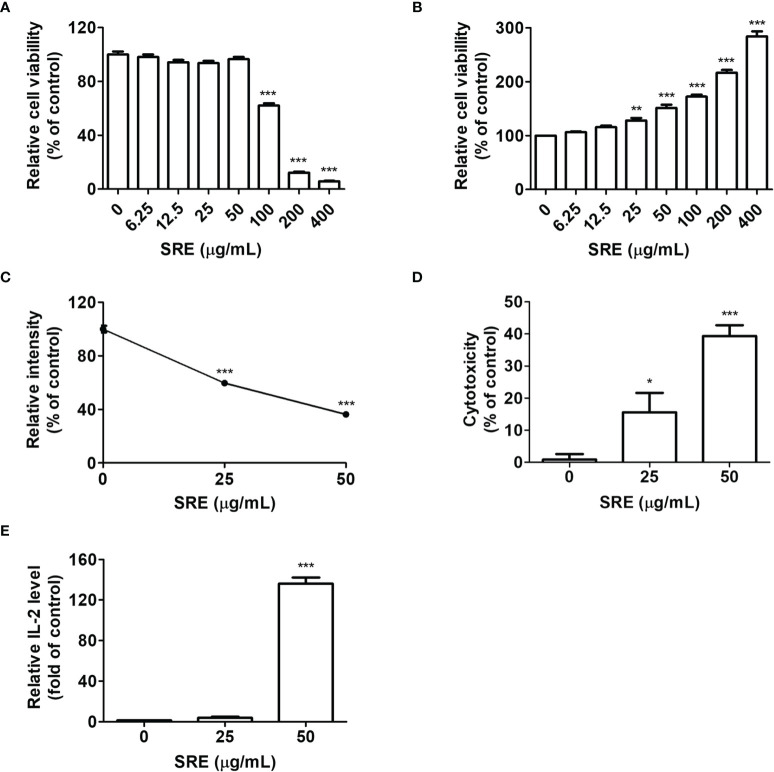
SRE-induced activation of T cells and cytotoxic effect of T cell-mediated cancer cells. **(A, B)** The cell viability was performed using the CCK-8 assay. Splenocytes were isolated from hPD-L1 MC38 cell-bearing hPD-1 knockin mice. Murine CRC hPD-L1 MC38 cells **(A)** and hPD-1 mice splenocytes **(B)** were treated with SRE for 72 hours. **(C)** Cocultured hPD-L1 MC38 cell viability tested by crystal violet staining; **(D)** Lactate dehydrogenase (LDH) released by damaged cells, detected *via* LDH cytotoxicity assay; **(E)** Relative interleukin-2 (IL-2) level, determined using the mouse IL-2 ELISA set. Data are presented as the mean ± SD. *p < 0.05, **p < 0.01, and ***p < 0.001 compared to the control.

We hypothesized that SRE induces antitumor responses to blockade PD-1/PD-L1, as T cell activation by medicinal herb extracts has previously been reported (20). To confirm the cytotoxicity of SRE against murine CRC hPD-L1 MC38 cells and hPD-L1 MC38 cell-bearing hPD-1 mice-isolated splenocytes, cells were incubated with various concentrations of SRE for 72 hours. SRE had no cytotoxic effect on hPD-L1 MC38 cells up to the concentration of 50 μg/mL (**Figure 3A**). CC_50_ concentrations of SRE on these cells were 114.94 ± 14.09 μg/mL. SRE increased splenocyte viability dose-dependently, suggesting that SRE can improve immune cell function (**Figure 3B**).

To elucidate whether T cells mediate SRE’s antitumor effect, coculture systems were conducted; hPD-1 splenocytes were used as effector cells, and hPD-L1 MC38 cancer cells known to express PD-L1 were used as target cells (21). At an effector cell-to-target cell ratio of 10:1, the cytotoxicity of hPD-L1 MC38 cells cocultured with hPD-1 splenocytes was gradually increased concentration-dependently (**Figures 3C, D**). CC_50_ concentrations of SRE on the hPD-L1 MC38 cells for 72 hours were 33.25 ± 7.20 μg/mL (**Figure 3C**). Moreover, released IL-2 increased dose-dependently, suggesting that T cells activated by SRE secrete IL-2 (**Figure 3E**). These results imply that SRE efficiently improved T cell immune function by blockading the PD-1/PD-L1 immune checkpoint pathway.

### SRE Activation of hPD-1^+^ Tumor-Infiltrating CD8^+^ T Cells From hPD-L1 MC38 Tumor Tissues

**Figure 4:**
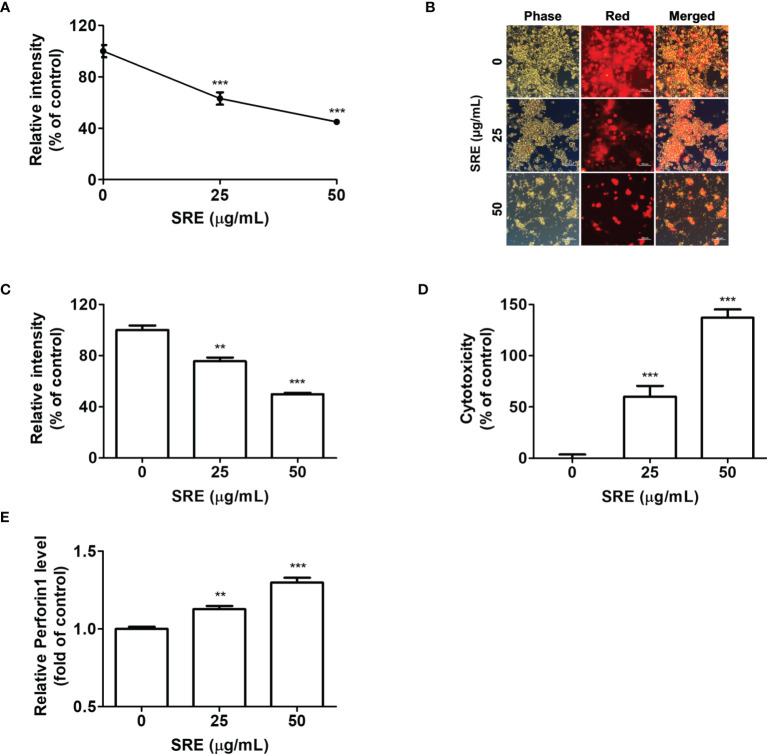
SRE elevated the activation of hPD-1^+^ CD8^+^ T cells and the CD8^+^ T cell-mediated killing effect on hPD-L1 MC38 cancer. **(A)** Cocultured hPD-L1 MC38 cell viability, tested by crystal violet staining. Cocultured hPD-L1 MC38 cells detected with fluorescence microscopy (× 200) **(B)** and determined by fluorescent-activated cell sorting analysis **(C)**. **(D)** LDH released from damaged cells; **(E)** Relative perforin 1 (PRF1) level, determined with use of the mouse PRF1 ELISA kit. Data are presented as the mean ± SD. **p < 0.01 and ***p < 0.001 compared to the vehicle group.

We confirmed that SRE suppressed hPD-L1 MC38 tumor growth in both *in vitro* and *in vivo* models by activating hPD-1 T cells. The additional delineation of the anticancer effect of tumor-infiltrating CD8^+^ T cells based on T cell activation by SRE treatment in CRC can also be targeted by immunotherapy. To further examine the cytotoxic role of tumor-infiltrating CD8^+^ T cells by SRE treatment, coculture systems were conducted using hPD-L1 MC38 cell-bearing hPD-1 mice-isolated CD8^+^ T cells as effector cells and hPD-L1 MC38 cancer cells as target cells. At an effector cell-to-target cell ratio of 5:1, the cytotoxicity of hPD-L1 MC38 cells cocultured with hPD-1 CD8^+^ T cells was gradually increased concentration-dependently (**Figure 4A**). CC_50_ concentrations of SRE on the hPD-L1 MC38 cells for 72 hours were 42.14 ± 8.17 μg/mL. Additionally, proliferation of hPD-L1 MC38 cells (labeled with CellTrace™ Far Red) was markedly reduced by SRE treatment (**Figures 4B, C**). Lactate dehydrogenase (LDH) release from the hPD-L1 MC38 cells, detected with use of LDH cytotoxicity assay, increased dose-dependently (**Figure 4D**). The released perforin 1 (PRF1) levels increased dose-dependently, suggesting that CD8^+^ T cells activated by SRE secrete PRF1 (**Figure 4E**). With the results of the effector cell-to-target cell coculture tests, this indicates that SRE efficiently enhanced tumor-infiltrating CD8^+^ T cell activation *via* blockade of PD-1/PD-L1 interaction in the CRC tumor microenvironment.

### Antitumor Effect of SRE in hPD-L1 MC38 Tumor-Bearing Humanized PD-1 Mouse Model

**Figure 5:**
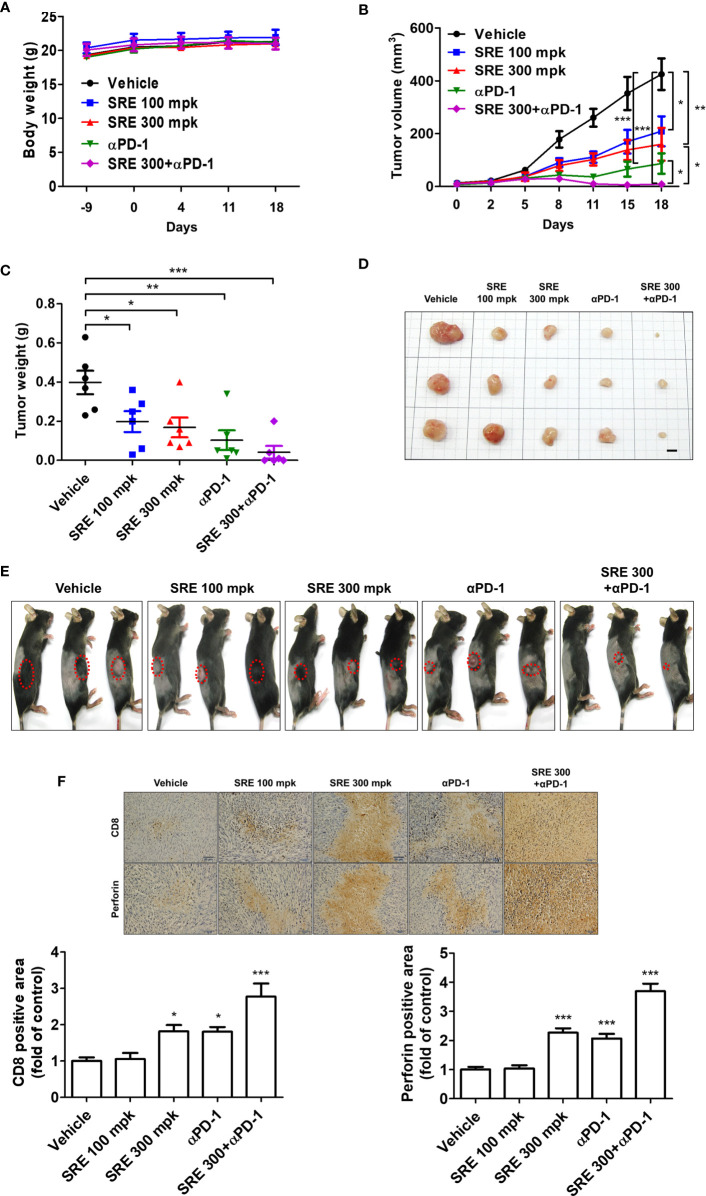
Sanguisorbae Radix extract reduced tumor growth in the hPD-L1 MC38 cell allograft hPD-1 mouse model. **(A)** Body weight (grams); **(B)** Tumor volume after 18 days; **(C)** Tumor weight after 18 days; **(D)** Images of tumor tissues (bar indicates 5 mm); **(E)** hPD-L1 MC38 tumor-bearing mice 18 days after treatment; **(F)** Representative microscopic images (×400) of CD8 and PRF1-positive area of tumor tissues calculated using immunohistochemical analysis. Data are presented as mean ± standard deviation. *p < 0.05, **p < 0.01, and ***p < 0.001 compared with the vehicle group.

We examined whether SRE could increase inhibition of tumor growth induced by activated T cells on the hPD-L1 MC38 cell-bearing humanized PD-1 knockin mouse model. The hPD-L1 MC38 murine CRC cells were injected into the allograft mice model. After 10 days, tumor volumes reached 20 mm^3^ and mice were randomized into groups of six animals per group to investigate the antitumor effect *in vivo*, of treatment with SRE and anti-PD-1 antibodies. SRE significantly inhibited hPD-L1 MC38 allograft tumor growth in a dose-dependent manner without affecting body weight, as observed by decreased tumor volume and weight (**Figures 5A–E**). Notably, the combination of SRE and anti-PD-1 antibodies synergistically suppressed tumor growth to a greater extent than did either agent alone. SRE and anti-PD-1 antibodies did not change aspartate aminotransferase (AST), alanine aminotransferase (ALT), BUN, or creatinine levels in mice serum (**Table 1**).

**Table 1:** AST, ALT, BUN, and creatinine levels in SRE-treated mice serum.

	AST (IU/L)	ALT (IU/L)	BUN (mg/dL)	Creatinine (mg/dL)
Vehicle	97.66 ± 9.33	28.00 ± 9.03	32.33 ± 1.41	0.1
SRE 100 mpk	90.00 ± 30.54	19.33 ± 3.93	28.66 ± 2.50	0.1
SRE 300 mpk	74.00 ± 23.01	20.66 ± 4.67	35.66 ± 3.75	0.1
αPD-1	71.33 ± 16.47	23.33 ± 5.88	35.40 ± 5.60	0.1
SRE 300+αPD-1	82.00 ± 32.56	25.33 ± 8.71	30.53 ± 4.78	0.1

Values are presented as the mean ± SD of six mice. ALT, alanine aminotransferase; AST, aspartate aminotransferase; BUN, blood urea nitrogen.

Immunohistochemistry staining showed that SRE and anti-PD-1 antibodies increased CD8 (a marker of CD8^+^ T cells) and PRF1 granule exocytosis involved in cytotoxic T cell-mediated tumor cell death in the tumor tissues (**Figure 5F**). These investigations demonstrated that the combination of SRE and anti-PD-1 antibodies successfully suppressed tumor growth by improving CD8^+^ T cell infiltration with antitumor immunity in the humanized PD-1 mouse model.

## Discussion

Research into antitumor immunity through immune checkpoint blockades with an anti-PD-1/PD-L1 interaction for the treatment of patients with CRC is notable (7). Some CRCs are characterized by advanced/metastatic solid malignancies, TIL enrichment, and upregulated PD-L1 expression within the tumor microenvironment (22). Pembrolizumab, as an anti-PD-1, has been approved for immunotherapy of CRC with microsatellite instability or mismatch-repair deficiency (23). However, clinical agents have a large molecular weight, which is slowly absorbed, and cause various side effects (3). To overcome the shortcomings of clinical therapies in the CRC treatment, we tried to use an oriental medicinal herb, SRE, which has a low molecular weight.

Although several reports have shown SRE use in treatment of human CRC, breast cancer, and prostate cancer (18, 24, 25), SRE’s anticancer activities *via* mediation of T cells had not been defined. This study demonstrated the experimental evidence on antitumor immunity by improving T cell activity with SRE. In the present study, we discovered the ability of SRE to enhance T cell functionality *via* PD-1/PD-L1 interaction blockade using PD-1/PD-L1 ELISA-binding assay and PD-1/PD-L1 blockade bioassay. Additionally, we found a difference, in that SRE activates tumor-infiltrating CD8^+^ T cells and kills CRC cells in the tumor microenvironment. In particular, this study confirmed that CD8^+^ T cells activated by SRE secrete PRF1 to kill CRC cells.

Nontoxic dose of SRE in splenocyte-tumor coculture systems, we tested the cytotoxicity of SRE in hPD-L1 MC38 cells and humanized PD-1 mice-isolated splenocytes (**Figure 3**). In line with a previous study (26), SRE over 100 μg/mL is cytotoxic to a liver cancer cell line; however, 50 μg/mL of SRE has a 40% cytotoxicity and extremely released IL-2 in splenocyte-tumor coculture systems. Interestingly, the more an SRE dose increases up to 400 μg/mL, the more proliferation of splenocytes increases and tumor proliferation decreases. There are reports of SRE inducing immunomodulatory effects including inflammatory responses and cytokine production (27, 28). Our findings suggest that SRE would not only have a cytotoxic effect on CRC cells but also promote T cell immunity and have anticancer effects *via* inhibition of PD-1/PD-L1 interaction in splenocyte-tumor coculture systems.

The humanized immune checkpoint mice are carefully designed as immuno-oncology mouse models for reliable *in vivo* evaluation and validation of checkpoint blockers drugs and their combination with other antitumor drugs (29). According to previous studies, although there are structural similarities between human and mouse PD-L1 proteins, there are significant differences in the druggability of these two proteins (30). In line with reported results, our study showed that small molecules, peptides, and some human anti-PD-L1 antibodies bound to human PD-L1, but not to mouse PD-L1. In addition, there were no effects observed in MC38 tumor-bearing immunocompetent mice treated with human anti-PD-L1 antibodies. Moreover, the MC38 used *in vivo* in this study is derived from tumors induced by carcinogen and established in C57BL/6 mice and represents a microsatellite-unstable CRC cell line (31). We have previously reported the PD-1/PD-L1-inhibiting abilities of medicinal herbs including *Salvia plebeia* alone and *Rubus coreanus* alone *in vivo* (20, 32). Here, we found that SRE in combination with anti-PD-1 antibodies *in vivo* is a potent PD-1/PD-L1 inhibitor. We assessed the synergistic effect of the combination of SRE and anti-PD-1 antibodies using humanized PD-1 knockin mice and humanized PD-L1 MC38 tumor cells and successfully established a CRC immunotherapy. As shown in **Figure 5**, the antitumor effect of SRE was higher in a dose of 300 mg/kg group than in a 100 mg/kg group; the effect of SRE in combination with anti-PD-1 antibodies was considerably greater than 300 mg/kg SRE alone or anti-PD-1 antibodies alone. In addition, combination of SRE and anti-PD-1 antibodies remarkably increased infiltration of CD8^+^ T cells in tumor tissues more than either 300 mg/kg SRE alone or anti-PD-1 antibodies alone, as well as increasingly released PRF1 granules of tumor-infiltrating CD8^+^ T cells *via* PD-1/PD-L1 blockade in the tumor microenvironment. The combination of SRE and anti-PD-1 antibodies is thus expected to be a workhorse for preclinical investigational studies in patients with CRC, supporting the validity of cancer immunotherapies.

Biochemical analysis also confirmed that the indicated dosages of SRE and anti-PD-1 antibodies have no significant toxic effect on the liver or kidneys in humanized PD-1 mice with humanized PD-L1 MC38 tumors (**Table 1**). In a Mongolian gerbil, daily oral administration of SRE at a dose of 400 mg/kg for 28 days, no gross histological changes were found (33). Moreover, the no changes were found in the relative weights of liver and kidney in the gerbil, or in AST and ALT in high-fat–diet-induced obese C57BL/6J mice after 8 weeks’ treatment with 200 mg/kg/day SRE extracted with 50% ethanol (34). In the current study, 100 mg/kg/day of SRE in mice is equivalent to 480 mg/kg/day for a 60 kg human. *S. officinalis* is safe enough to be approved for use as a food ingredient, and the root of the plant, SR, is considered nontoxic.

This study provides evidence that SRE enhanced the potential antitumor immunologic response by regulating the PD-1/PD-L1 axis for the treatment of CRC. Additionally, it established that SRE in combination with anti-PD-1 antibodies has strong CD8^+^ T cell-mediated antitumor activity in an hPD-L1 MC38 cell-bearing hPD-1 knockin mouse model. Recently, because ICIs alone have shown limited efficacy in patients with CRC, combinations of ICIs with other agents, such as anti-PD-L1 antibodies or chemotherapy, are being tested in patients with CRC in clinical studies (10); however, applied combination therapies have several adverse effects, including toxicity, resistance, and side effects (3). To improve upon the clinical combination therapies, we used a combination of clinical antibodies with SRE, with easy dose adjustment and fast absorption in a humanized mice model.

Among the ingredient compounds of SR, several phenolic compounds have been reported as antitumor agents (19). Several other chemical constituents, including tannin, triterpenoid, flavonoids, and triterpene glycoside, have been found in *S. officinalis* roots (35–37). It is not clear which components are responsible for PD-1/PD-L1 blockade and the subsequent antitumor effect of CRC. Further studies are needed to elucidate, which molecules from SR inhibit the PD-1/PD-L1 interaction.

## Conclusions

We demonstrated that SRE inhibits PD-1/PD-L1 interaction and suppresses growth of CRC cells by enhancing T cell functional activity. In addition, we established that SRE in combination with anti-PD-1 antibodies significantly reduces CRC tumor growth *via* tumor-infiltrating CD8^+^ T cell activities in an hPD-L1 MC38 cell-bearing hPD-1 knockin mouse model. From these results, we suggest that SRE is a novel inhibitor of PD-1/PD-L1 interaction and, in combination with antibody drugs, may provide a new strategy for weakening CRC mortality as an antitumor immunity drug.

## Data Availability Statement

The original contributions presented in the study are included in the article/supplementary material. Further inquiries can be directed to the corresponding authors.

## Ethics Statement

The animal study was conducted according to the guidelines of the Institutional Animal Care and Use Committee (IACUC) of the Korea Institute of Oriental Medicine (KIOM), and approved by the IACUC of the KIOM (approval number: KIOM-D-20-073).

## Author Contributions

E-JL, J-GC, and H-SC designed the study, conducted the experiments and wrote the manuscript. JK, TK, Y-JK, MP, CJ, YP, WL, and YK performed the experiments. H-SC supervised the research and wrote the manuscript. All authors contributed to the article and approved the submitted version.

## Funding

This research was supported by the Korea Institute of Oriental Medicine (KIOM) Grant number KSN2021230 provided by the Ministry of Science and ICT, Republic of Korea.

## Conflict of Interest

The authors declare that the research was conducted in the absence of any commercial or financial relationships that could be construed as a potential conflict of interest.

## Publisher’s Note

All claims expressed in this article are solely those of the authors and do not necessarily represent those of their affiliated organizations, or those of the publisher, the editors and the reviewers. Any product that may be evaluated in this article, or claim that may be made by its manufacturer, is not guaranteed or endorsed by the publisher.
